# ADORA2A C Allele Carriers Exhibit Ergogenic Responses to Caffeine Supplementation

**DOI:** 10.3390/nu12030741

**Published:** 2020-03-11

**Authors:** Jozo Grgic, Craig Pickering, David J. Bishop, Juan Del Coso, Brad J. Schoenfeld, Grant M. Tinsley, Zeljko Pedisic

**Affiliations:** 1 Institute for Health and Sport (IHES), Victoria University, Melbourne 3011, Australia; david.bishop@vu.edu.au (D.J.B.); zeljko.pedisic@vu.edu.au (Z.P.); 2 Institute of Coaching and Performance, School of Sport and Wellbeing, University of Central Lancashire, Preston PR1 2HE, UK; craigpickering1014@hotmail.com; 3 School of Medical and Health Sciences, Edith Cowan University, Joondalup, Perth, WA 6027, Australia; 4 Centre for Sport Studies, Rey Juan Carlos University, C/Camino del Molino, s/n, 28943 Fuenlabrada, Spain; juan.delcoso@urjc.es; 5 Department of Health Sciences, Lehman College, Bronx, NY 10468, USA; bradschoenfeldphd@gmail.com; 6 Energy Balance & Body Composition Laboratory, Department of Kinesiology & Sport Management, Texas Tech University, Lubbock, TX 79424, USA; Grant.Tinsley@ttu.edu

**Keywords:** caffeine, ergogenic aid, genetics, mean velocity

## Abstract

Caffeine’s ergogenic effects on exercise performance are generally explained by its ability to bind to adenosine receptors. *ADORA2A* is the gene that encodes A_2A_ subtypes of adenosine receptors. It has been suggested that *ADORA2A* gene polymorphisms may be responsible for the inter-individual variations in the effects of caffeine on exercise performance. In the only study that explored the influence of variation in *ADORA2A*—in this case, a common polymorphism (rs5751876)—on the ergogenic effects of caffeine on exercise performance, C allele carriers were identified as “non-responders” to caffeine. To explore if C allele carriers are true “non-responders” to the ergogenic effects of caffeine, in this randomized, double-blind study, we examined the acute effects of caffeine ingestion among a sample consisting exclusively of *ADORA2A* C allele carriers. Twenty resistance-trained men identified as *ADORA2A* C allele carriers (CC/CT genotype) were tested on two occasions, following the ingestion of caffeine (3 mg/kg) and a placebo. Exercise performance was evaluated with movement velocity, power output, and muscle endurance during the bench press exercise, countermovement jump height, and power output during a Wingate test. Out of the 25 analyzed variables, caffeine was ergogenic in 21 (effect size range: 0.14 to 0.96). In conclusion, *ADORA2A* (rs5751876) C allele carriers exhibited ergogenic responses to caffeine ingestion, with the magnitude of improvements similar to what was previously reported in the literature among samples that were not genotype-specific. Therefore, individuals with the CT/CC genotype may still consider supplementing with caffeine for acute improvements in performance.

## 1. Introduction

The effects of caffeine on exercise have received substantial attention in the scientific literature [1,2,3,4,5,6,7,8]. Currently, it is well established that acute ingestion of caffeine doses in the range from 2 to 6 mg per kilogram of body mass enhances exercise performance [1,2,3,4,5,6,7,8]. Caffeine’s ergogenic effects are apparent in different components of exercise. For example, a recent umbrella review reported that caffeine ingestion enhances muscle strength and endurance, aerobic endurance, power output, and jumping performance [3]. Even though research indicates that caffeine ingestion may be acutely ergogenic for a wide range of exercise tasks, between-person variability in responses to this dietary supplement seems substantial [9,10]. The ergogenic effects of caffeine are generally explained by its interaction with adenosine A_1_, A_2A_, and A_2B_ receptors [11,12]. Adenosine concentrations in the brain progressively increase during waking hours, resulting ultimately in sensations of fatigue; the concentrations of adenosine also decrease during sleep. Caffeine’s molecular structure is similar to that of adenosine. Therefore, after ingestion, caffeine binds to adenosine receptors, subsequently resulting in reduced fatigue, increased vigilance, and ergogenic effects on exercise performance [11,12].

Researchers have suggested that the inter-individual variation in caffeine response may be due to polymorphisms within two genes, namely *CYP1A2* and *ADORA2A* [10]. Cytochrome P450 1A2 (an enzyme responsible for up to 95% of caffeine metabolism) is encoded by the *CYP1A2* gene [10]. A single nucleotide polymorphism rs762551 within *CYP1A2* affects the speed of caffeine metabolism. Specifically, individuals with the AA genotype are commonly classified as “fast caffeine metabolizers”, whereas C allele carriers (AC/CC genotypes) are considered to be “slow caffeine metabolizers”, respectively [13]. The influence of *CYP1A2* (rs762551) on the acute effects of caffeine supplementation on exercise performance has been explored in several studies [14,15,16,17,18,19,20,21,22,23]. However, the evidence in these studies remains inconsistent, with some reporting no effect of the polymorphism on the ergogenic effects of caffeine supplementation and others showing a modifying effect, but in different directions [14,15,16,17,18,19,20,21,22,23].

*ADORA2A* is the gene that encodes A_2A_ subtypes of adenosine receptors [24]. Previous research has suggested that this receptor represents the primary target of caffeine action in the central nervous system, and thus, polymorphic variations in the *ADORA2A* gene may impact the responses to acute caffeine ingestion [24]. The rs5751876 polymorphisms in the *ADORA2A* gene are comprised of a C-to-T substitution at nucleotide position 1083 (rs5751876) (also known as 1976C>T) [24]. Interestingly, as compared to TT homozygotes, *ADORA2A* C allele carriers have higher habitual caffeine consumption, which may suggest that these individuals need higher doses of caffeine to obtain a pharmacological effect [24].

Only one study has explored the influence of variation in this gene—in this case, a common polymorphism (rs5751876)—on the ergogenic effects of caffeine on exercise performance [25]. The study included 12 participants (6 TT homozygotes and 6 C allele carriers [i.e., CC/CT genotype]). These participants were untrained women who completed 20 min of cycling at a work rate eliciting 60% of VO_2peak_ followed by two 10-min cycling time trials. The exercise task was performed on two occasions, following the ingestion of 5 mg/kg of caffeine or a placebo. Results indicated that caffeine ingestion was ergogenic for TT homozygotes but not for C allele carriers. Based on this study, C allele carriers were identified as “non-responders” to caffeine [25].

Given the limited data on this topic, the aim of this study was to explore the influence of *ADORA2A* (rs5751876) on the acute effects of caffeine supplementation on exercise performance, by using exercise tests for which caffeine had previously been shown to be ergogenic [3].

## 2. Materials and Methods

### 2.1. Experimental Design

In this double-blind, randomized, crossover trial, all participants attended four laboratory sessions (in the morning hours between 07:00 to 12:00 h) that were from 4 to 7 days apart. The first two sessions consisted of familiarization with the exercise protocol. The third and fourth sessions were the main sessions. Twenty-four hours before the main trials, participants were asked the following: (a) to avoid any intense exercise; (b) to track their energy and macronutrient intake; and (c) to refrain from caffeine intake after 6 pm on the day before testing. The participants performed the two main sessions in a fasted state (overnight fast). Caffeine and placebo supplementation was provided on different days. Caffeine (Pure Lean Nutrition, Melbourne, Australia) was administered in a gelatin capsule with a dose of 3 mg/kg of body mass, while the placebo gelatin capsule contained 3 mg/kg of body mass of dextrose. All capsules were of identical appearance. Placebo and caffeine powders were weighed using a high precision electronic digital scale (Precisa, XT 120A, Dietikon, Switzerland) and then packaged into capsules. Capsules were prepared in the laboratory by an experienced researcher while other researchers performed the blinding. Capsules were ingested 60 min before the start of the exercise session under the supervision of the research staff, as in previous research [1,26,27]. The participants’ genotype was determined using a buccal swab. Ethical approval was requested and granted from the Victoria University Human Research Ethics Committee (number: HRE19-019), and every participant signed an informed consent form.

### 2.2. Participants

The study included a sample of 22 resistance-trained men, defined herein as having a minimum of six months of resistance training experience with a minimum weekly training frequency of two times on most weeks. Exclusion criteria were the existence of any health limitations and prior use of anabolic steroids (self-reported). All participants completed all sessions with no injuries or adverse events. Participants’ characteristics are presented in Table 1.

### 2.3. Exercise Protocol

Exercises involving the upper body were performed prior to those that predominately activated the lower body, to avoid any transfer of muscle fatigue from one exercise task to another. At the beginning of the exercise protocol, the participants performed the bench press exercise with different loads (i.e., 25%, 50%, 75%, and 90% of one-repetition maximum (1RM)—performed in that order) [28]. 1RM was established during the first familiarization session. At each respective load, the participants performed two sets of one repetition, separated by a 3-min rest interval. The better repetition at each load was used for the analysis. The eccentric phase lasted 2 s, there was no pause at the bottom phase, and the concentric action was performed with maximal velocity. Mean power (W), mean concentric velocity (m/s), peak power (W), and peak concentric velocity (m/s) were measured for each repetition using the GymAware linear position transducer device (GymAware Power Tool, Kinetic Performance Technologies, Canberra, Australia) that was attached to the barbell.

After the second set that was performed with 90% of 1RM, the participants were provided with five minutes of rest. Then, we tested upper-body muscular endurance with a task that involved performing repetitions to momentary muscular failure in the bench press exercise with a load of 85% of 1RM. In this test, we collected data on the total number of repetitions, as well as power and velocity output of each repetition using the linear position transducer attached to the barbell. The tempo was the same as in the previous task. For the statistical analysis, we compared the total number of repetitions between the placebo and caffeine conditions. In addition, to explore the “quality” of performed repetitions, we matched the number of repetitions between the placebo and caffeine conditions and examined their average power and velocity. For example, one participant performed 7 and 8 repetitions following the ingestion of the placebo and caffeine, respectively. In this case, we only examined the velocity and power of the first 7 repetitions in both conditions.

After the muscular endurance test, the participants rested for three minutes. Then the participants performed a short warm-up consisting of one minute of light running, followed by ten bodyweight squats. After the warm-up, participants performed a countermovement jump (CMJ) without an arm swing on a force platform (400S Isotronic Fitness Technology, Skye, Australia). The participants positioned themselves in an upright starting position and received commands from the computer software associated with the force platform that was positioned in front of the platform. This software visually counted down, “3, 2, 1” and provided “Set” and “Go” commands. After the “Go” command, the participants had five seconds to complete the jump. The participants performed a fast knee flexion (where their lowest position was a semi-squat position) [29,30]. Immediately after reaching this point (i.e., no pause at the bottom phase), the participants rapidly extended the hip, knee, and ankle joints with prior instructions to jump as quickly and “explosively” as possible to achieve maximal vertical jump height [29,30]. A total of three attempts was provided with one minute of rest between them. The best jump was used for the analysis. The outcome in the CMJ test was vertical jump height.

After the CMJ, the participants rested for three minutes. Then, the participants performed the Wingate test on an Excalibur Sport Cycle Ergometer (Lode, Groningen, The Netherlands). The Wingate test started with a 5-min warm-up consisting of pedaling at 100 W at 60–80 rpm [31]. Following the warm-up, participants performed a 30-s “all-out” sprint on the bike. The flywheel resistance was set at 0.075 Nm/kg. The participants were instructed to remain seated during the 30-s sprint.

### 2.4. Assessment of Blinding

In both main trials (i.e., caffeine and placebo), before and after the testing session, participants responded to the following question: “Which supplement do you think you have ingested?” [32]. This question was used to explore the effectiveness of the blinding and had three possible responses: (a) “caffeine”, (b) “placebo”, and (c) “I do not know” [32]. If the participants responded with “a” or “b”, they were also asked to state the reason for choosing their respective response.

### 2.5. Genetic Testing

Genetic testing was performed using a commercially available testing kit from DNAfit Life Sciences. The procedure used for genetic testing is explained in detail elsewhere [33]. Briefly, the buccal swab sample was collected using OCR-100 kits by DNAGenotek. For the analysis, these samples were sent to IDna Genetics Laboratory (Norwich, UK). DNA was: (a) extracted and purified using the Isohelix Buccalyse DNA extraction kit BEK-50 (Kent, UK); and (b) amplified using polymerase chain reaction (PCR) on an ABI 7900 real-time thermocycler (Applied Biosystem, Waltham, MA, USA). The collected samples were analyzed for the *ADORA2A* (rs5751876) single-nucleotide polymorphism. Genotype analyses were performed after the exercise performance data collection was finalized. Therefore, researchers and participants were blinded to genotype variations of the sample during the exercise performance data collection.

### 2.6. Statistical Analysis

Two participants who were *ADORA2A* TT homozygotes were excluded, leaving a total of 20 C allele carriers (CC and CT) in the analysis. One-way repeated-measures analysis of variance (ANOVA) was used to analyze the exercise performance data. Relative effect sizes (and their 95% confidence intervals; 95% CI) were expressed using Hedges’ g for repeated measures. The effect sizes were classified as follows: trivial (<0.20); small (0.20–0.49); moderate (0.50–0.79); and large (≥0.80). The effectiveness of blinding was examined using the Bang’s Blinding Index, as explained elsewhere [29]. All analyses were performed using the Statistica software (version 13.0; StatSoft; Tulsa, OK, USA). The significance level was set at *p* < 0.05. 

## 3. Results

### 3.1. Exercise Performance

**Figure 1:**
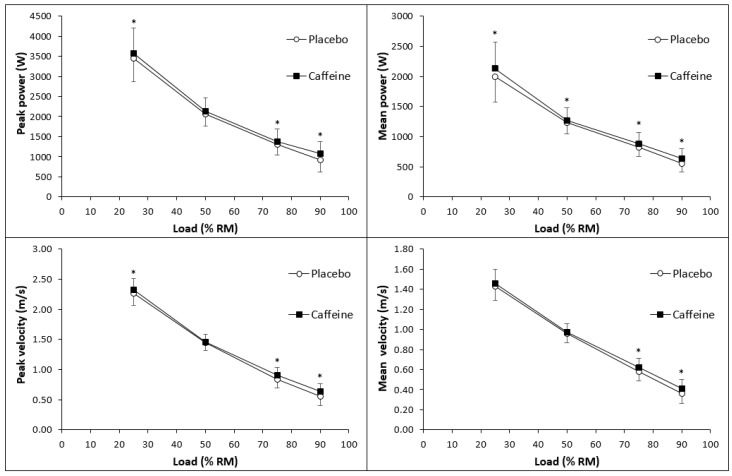
The effects of caffeine vs. placebo on peak power (upper left section), peak velocity (lower left section), mean power (upper right section), and mean velocity (lower right section) in the bench press with 25%, 50%, 75%, and 90% of one repetition maximum (1RM). Data are presented as mean ± standard deviation. * denotes significant differences between the conditions.

For movement velocity and power, we found significant effects of caffeine ingestion for all outcomes except for mean velocity at 25% of 1RM, and mean velocity, peak power, and peak velocity at 50% of 1RM (Figure 1). The significant effect sizes ranged from 0.16 to 0.53. For muscular endurance, we found significant effects of caffeine ingestion on the total number of performed repetitions and the quality of repetitions when matched for repetitions between the conditions. Here, the effect sizes ranged from 0.27 to 0.96 (Table 2). We also found a significant effect of caffeine ingestion on vertical jump height with an effect size of 0.13. For power output in the Wingate test, we found significant effects of caffeine ingestion on peak, mean, and minimum power. The effect sizes ranged from 0.34 to 0.41.

### 3.2. Assessment of Blinding

Before the start of the exercise session, 50% and 65% of the participants correctly guessed (beyond chance) the placebo and caffeine conditions, respectively. After finishing the exercise session, 65% and 75% of the participants correctly guessed the placebo and caffeine conditions beyond chance, respectively. Participants who correctly identified caffeine reported “feeling more energized” and/or “more alert”, or they associated the improvements in exercise performance with caffeine ingestion. 

## 4. Discussion

The main finding of this study is that caffeine ingestion may be ergogenic for *ADORA2A* (rs5751876) C allele carriers in a range of exercise performance outcomes. Therefore, these results do not support the theoretical supposition that *ADORA2A* C allele carriers do not experience improvements in exercise performance following caffeine ingestion.

Our findings are not in accord with the Loy et al. [25] study, which proposed that *ADORA2A* C allele carriers do not experience an ergogenic response to caffeine supplementation. The main differences between our study and Loy et al. [25] are the sex of the participants and the exercise tests employed. Specifically, we included male participants, whereas Loy and colleagues included females. Therefore, it may be that female *ADORA2A* C allele carriers experience a different response to caffeine ingestion as compared to their male counterparts. However, this explanation is perhaps less plausible because recent evidence suggests that female and male participants experience similar ergogenic responses to caffeine ingestion in aerobic-, anaerobic- and strength-based exercise tasks [34,35,36]. Importantly, the present study and the work by Loy et al. [25] also differed in the selection of performance tests; while we assessed changes in power, muscular endurance, and sprinting performance, Loy and colleagues focused on aerobic endurance. It may be that caffeine affects performance in these components of exercise performance through different mechanisms. The possible impact of genetic variations may be more expressed in some tests and less in others. Given the scarce evidence on the influence of polymorphisms in *ADORA2A* on the individual variation in responses to caffeine, this topic certainly requires further research. Finally, given that we report here that *ADORA2A* C allele carriers improve performance following caffeine ingestion, this may suggest that other genotypes that were not tested herein (e.g., CYP1A2 AA and AC/CC genotypes) are more important for the individual responses to caffeine ingestion.

Interestingly, the effects of caffeine on exercise performance in this study were very similar in size to the effects previously reported in the literature. For example, the increases in muscular endurance in our study are similar to the performance benefits of caffeine recorded in a previous study that included individuals with *CYP1A2* (rs762551) AA genotype—which are suggested to experience the most profound ergogenic benefits of caffeine [22]. Furthermore, the increases in movement velocity, vertical jump height, and power output in the Wingate test are comparable to the improvements reported in meta-analyses of these outcomes among samples that were not genotype-specific [5,7,37]. For example, one meta-analysis [7] reported that caffeine ingestion acutely enhanced Wingate peak power by an effect size of 0.27 (95% CI: 0.08, 0.47), which is very similar to the effect size of 0.37 (95% CI: 0.21, 0.55) observed in this study. 

### Strengths and Limitations

The main strength of the present study was the use of a randomized, double-blind study design, which is identified as the gold standard in sports nutrition [38]. Additionally, the strength of the present study was in the use of exercise tests for which caffeine had been shown to be ergogenic. 

The main limitation of this study was that 50% to 75% of the participants were able to identify caffeine and placebo conditions beyond chance. However, these results were not a likely explanation of the differences in findings between our study and the Loy et al. [25] study, given that the majority of participants (>75%) in the Loy et al. study were able to guess the content of the capsules correctly. Additionally, given the small number of *ADORA2A* TT homozygotes in our sample, we could not assess whether TT homozygotes experience different responses to caffeine ingestion compared with C allele carriers, an area that should be explored in future research. The low number of participants classified as TT homozygotes could be explained by the estimate that around 85% of the population possess the CC/CT genotype at rs5751876 [39].

Finally, to avoid any potential confounding by prior food and caffeine ingestion [40,41], we opted to test the participants in a fasted state. This needs to be acknowledged as a limitation given that caffeine supplementation and exercise in a fasted state is likely not a “real-life” practice of many individuals, and is not in line with the current sports nutrition recommendations [42]. Future studies may consider further exploring this topic by using caffeine supplementation protocols that mirror those more commonly observed in practice.

## 5. Conclusions

Our findings suggest that *ADORA2A* (rs5751876) C allele carriers respond positively to caffeine supplementation. Therefore, individuals with the CT/CC genotype may still consider supplementing with caffeine for acute improvements in performance. Future research is needed to explore if *ADORA2A* TT homozygotes experience different responses to caffeine supplementation than C allele carriers.

## Figures and Tables

**Table 1:** Characteristics of the participants.

Variable	Mean ± Standard Deviation
Age (years)	29.3 ± 4.8
Body mass (kg)	80.3 ± 11.2
Height (cm)	183.1 ± 5.9
1RM in the bench press (normalized per body mass)	1.1 ± 0.2
Habitual caffeine intake (mg/day)	143 ± 113

1RM: one repetition maximum.

**Table 2:** Effects of caffeine ingestion on performance in the muscular endurance test, countermovement jump, and Wingate: results from a series of one-way repeated measures analyses of variance.

Variable	Placebo	Caffeine	Hedges’ g and 95% CI	*p*-Value
Muscular endurance test				
Maximum repetitions at 85% 1RM	6.9 ± 2.2	8.2 ± 2.1	0.58 (0.29, 0.91)	<0.001
Mean power matched for repetitions (W)	418 ± 116	492 ± 138	0.56 (0.32, 0.83)	<0.001
Mean velocity matched for repetitions (m/s)	0.27 ± 0.05	0.32 ± 0.05	0.96 (0.58, 1.41)	<0.001
Peak power matched for repetitions (W)	669 ± 250	740 ± 258	0.27 (0.14, 0.42)	<0.001
Peak velocity matched for repetitions (m/s)	0.41 ± 0.08	0.46 ± 0.07	0.64 (0.38, 0.94)	<0.001
CMJ				
Vertical jump height (cm)	35.0 ± 6.1	35.8 ± 5.9	0.13 (0.02, 0.25)	0.034
Wingate test				
Peak power in the Wingate test (W)	859 ± 237	948 ± 229	0.37 (0.21, 0.55)	<0.001
Mean power in the Wingate test (W)	598 ± 101	634 ± 100	0.34 (0.17, 0.54)	<0.001
Minimum power in the Wingate test (W)	349 ± 103	392 ± 96	0.41 (0.07, 0.78)	0.020

1RM: one repetition maximum: CMJ: countermovement jump; CI: confidence interval.
